# A Novel Gesture Recognition System Based on CSI Extracted from a Smartphone with Nexmon Firmware

**DOI:** 10.3390/s21010222

**Published:** 2020-12-31

**Authors:** Tao Li, Chenqi Shi, Peihao Li, Pengpeng Chen

**Affiliations:** 1 School of Computer Science and Technology, China University of Mining and Technology, Xuzhou 221116, China; li_t@cumt.edu.cn (T.L.); shichenqi@cumt.edu.cn (C.S.); lph@cumt.edu.cn (P.L.); 2 China Mine Digitization Engineering Research Center, Ministry of Education, Xuzhou 221116, China

**Keywords:** gesture recognition, WiFi, channel state information, nexmon firmware

## Abstract

In this paper, we propose a novel gesture recognition system based on a smartphone. Due to the limitation of Channel State Information (CSI) extraction equipment, existing WiFi-based gesture recognition is limited to the microcomputer terminal equipped with Intel 5300 or Atheros 9580 network cards. Therefore, accurate gesture recognition can only be performed in an area relatively fixed to the transceiver link. The new gesture recognition system proposed by us breaks this limitation. First, we use nexmon firmware to obtain 256 CSI subcarriers from the bottom layer of the smartphone in IEEE 802.11ac mode on 80 MHz bandwidth to realize the gesture recognition system’s mobility. Second, we adopt the cross-correlation method to integrate the extracted CSI features in the time and frequency domain to reduce the influence of changes in the smartphone location. Third, we use a new improved DTW algorithm to classify and recognize gestures. We implemented vast experiments to verify the system’s recognition accuracy at different distances in different directions and environments. The results show that the system can effectively improve the recognition accuracy.

## 1. Introduction

Human gesture recognition and activity recognition are gradually becoming prominent promoters of human-computer interaction technology development. Traditionally, gesture recognition is usually implemented using technologies such as depth and infrared cameras [1,2,3] or wearable devices [4,5,6]. However, these solutions require significant overhead and non-negligible costs when deployed. The camera-based method mainly uses computer vision processing technology limited by the line of sight and light intensity, requires high surrounding environmental conditions, and has the risk of privacy leakage. Although the wearable device-based method can achieve high accuracy, the wearable sensing device needs to be attached to the user’s hand or body, which may cause inconvenience in some cases and requires higher additional costs. To overcome these limitations, WiFi-based gesture recognition systems have emerged in recent years [7,8,9,10,11,12,13].

Existing WiFi-based gesture recognition research can be roughly divided into two categories. One is based on Channel State Information (CSI), which is extracted from computers equipped with Intel 5300 network cards or Atheros 9580 network cards [14,15]. The other is based on a Received Signal Strength Indicator (RSSI), which can be extracted directly from a smartphone.

In the former, because the Intel 5300 network card can only collect 30 subcarriers, which cannot meet the fine-grained recognition of gestures, so most of them use multiple links to increase the number of subcarriers for CSI acquisition. Multiple transmitters or receivers with multiple antennas must be deployed. In addition, the person performing gesture recognition needs to prepare a laptop in advance and walking to a specific range near the laptop for gesture recognition. On account of the hard moving of the transceiver, the difficulty of gesture recognition is significantly increased when the relative position of the person and the transceiver is changed, and the recognition accuracy will be greatly reduced.

In the latter, the mobility of the smartphone can ensure the relative position of the personnel and the receiving terminal to a certain extent, which can better solve these problems. However, due to the CSI at the bottom of the smartphone cannot be obtained, the existing gesture recognition based on smartphone WiFi exploits RSSI. For example, WiGest [16] uses the influence on the RSSI of gestures close to the mobile receiver, realize gesture recognition, and then maps gestures to different applications operations. However, limited by the low dimensionality and coarse granularity of RSSI, in order to eliminate the influence caused by different distances between people and equipment, WiGest needs to cooperate with computer-side CSI to ensure accuracy. These limitations make these methods difficult to deploy into a practical and user-friendly system for gesture recognition. As RSSI is rough and susceptible to environment and distance, it is not easy to achieve stable high precision for fine gesture recognition.

To break through these limitations, some researchers [17,18] try to realize cross-domain gesture recognition by using transfer learning and antagonistic learning. However, these schemes basically require additional data collection and model retraining each time when a new target domain is added to the recognition model. Moreover, Widar 3.0 [19] realizes cross-domain gesture recognition in different locations and environments by deriving and estimating the speed profile of gestures at lower signal levels. However, such a scheme requires the deployment of six transceivers in a 2×2-m area to achieve higher accuracy.

In response to these existing problems, we hope to establish an easy-to-deploy, low-cost gesture recognition system that can be deployed on smartphones and only requires one transmitter and one receiver, leveraging the convenient mobility of smartphones to better adapt to the environment and changes in personnel positions. However, to implement such a system, we face three main technical challenges. First, the existing smartphones only can obtain RSSI and cannot extract CSI. The coarse-grained RSSI cannot guarantee the accuracy of gesture recognition. Second, the interference of the environment and personnel will affect the accuracy of the system. Finally, when different users and even the same users perform the same action, there are individual differences in gesture speed, action amplitude, and duration.

To address these challenges, a novel gesture recognition system called MDGest based on smartphones is proposed. It only needs one transmitting end and one receiving end for rapid deployment. More importantly, it supports 802.11ac and allows for bandwidths of up to 80 MHz to extract 256 CSI subcarriers from smartphones exploiting nexmon firmware [20,21], which is more than the sum of the subcarriers collected by the Intel 5300 network card with eight links. Furthermore, in daily life, almost everyone carries a smartphone, so we can take out the smartphone for gesture recognition at any time, instead of preparing a laptop in advance and walking near the laptop or bringing it to us for gesture recognition. To select subcarriers that better reflect the characteristics of gestures, we designed a key subcarrier screening algorithm. In response to environmental changes, we propose an environmental noise removal mechanism that uses a wavelet-based denoising method to filter out environmental noise while keeping the CSI mode due to the influence of gestures as much as possible. Aiming at the problems of individual diversity and inconsistency of gestures, the main pattern components of CSI are extracted and identified. An improved DTW algorithm is used to reduce the influence of smartphones distance and angle.

We implemented MDGest on a Google Nexus 5 phone and evaluated its performance with sixteen different users in three typical environments. Moreover, we conduct extensive experiments, including eight Los distances, eight gesture execution distances from the smartphone, four orientations, human interference situations, and comparisons with WiGest [16] and WiFinger [9].

In a nutshell, the main contributions of the paper are four-fold:

- firstly, we propose a novel gesture recognition system that extracts 256 CSI subcarriers from a smartphone in IEEE 802.11ac mode on 80 MHz bandwidth to implement gesture recognition using nexmon firmware.
- secondly, a method of extracting the main mode of CSI using cross correlation function and cross power spectral density is proposed, which can extract the time domain and frequency domain features of CSI.
- thirdly, we use a new improved DTW algorithm with higher computing efficiency, making our system have better adaptability to individual diversity and gesture inconsistency.
- fourthly, we conducted extensive experiments in three typical environments, with multiple participants under different conditions. The results show that MDGest achieves consistently high accuracies across different users in different environments and locations.

The rest of the paper is organized as follows. Section 2 introduce related works regarding wireless sensing system. Section 3 presents the details of the MDGest system. We implement the MDGest system and evaluate its performance in different scenarios in Section 4. In Section 5, we discuss some of the limitations of the MDGest system. Finally, we conclude the paper in Section 6.

## 2. Related Work

Our work is highly related to wireless human sensing technology. We break these tasks into two parts: wireless sensing systems, including WiFi-based indoor positioning, tracking, and general human activity recognition; gesture recognition systems, including gesture recognition, finger gesture recognition, and handwriting recognition systems.

### 2.1. Wireless Sensing System

WiFall uses features such as activity duration and rate of change in CSI values [22] to detect whether users fall down. E-eyes [23] is a pioneer in identifying human activities using commercial WiFi signal strength distribution and KNN. E-eyes generates histograms of CSI values and uses them as features to recognize gestures, such as brushing teeth, taking a bath, etc. HeadScan [24] and BodyScan [25] mounts the antenna on the users. HeadScan uses the average, median, and standard deviation of CSI values to identify gestures related to the mouth, such as coughing and eating. In terms of signals, existing methods extract various parameters of human reflection or occlusion signals, including DFS [26,27,28], ToF [29,30,31,32], AoA, AoD [29,32,33,34] and attenuation [35,36]. Depending on the type of equipment used, parameters with different accuracy and resolution can be obtained. WiDeo [32] customized full-duplex WiFi joint estimation of ToF and AoA of the main reflector. WiTrack [37] develops a wideband FMCW radar to estimate the ToFs of the reflected signals accurately, but special equipment is needed.

There are other systems that can recognize more subtle activities, such as EQ-Radio [38], which can infer the emotions of people from radio frequency signals reflected by the human body. WiSleep [39] supports non-invasive breathing monitoring on a single wireless link with a directional antenna. UbiBreathe [40] uses off-the-shelf WiFi devices to monitor breathing frequency and detect some forms of apnea. In addition, a system that uses radio wave signals [41] to learn the sleep stage has recently been proposed.

### 2.2. Gesture Recognition System

With the rapid development of WiFi-based activity recognition technology, gesture recognition has gradually become a research hotspot. WiFinger uses time series Discrete Wavelet Transform coefficients combined with CSI values to recognize gestures with different numbers of finger stretches and folds [9]. WiDraw tracks the hands of users by monitoring changes in signal strength arriving from different angles [7]. WiVi [33] uses the inverse synthetic aperture radar method to track human body movements, achieving the radar-like vision and simple through-wall gesture communication. WiSee [42] is a DopLink-based fine gesture recognition system [43,44] uses the Doppler frequency shift in the narrowband OFDM signal extracted to recognize nine different human gestures. WiKey [13] uses unique patterns in CSI to identify the keys of a finger.

Although these solutions provide high accuracy, they all require gesture recognition at a specified location that is relatively fixed to the transmit and receive links, and most solutions require special hardware or controlled environments. Therefore, the research of the environment and location-independent gesture recognition has gradually come into people’s field of vision. WiAG proposed a translation function to make gesture recognition independent of position and orientation [8]. Widar 3.0 [27] proposes a cross-domain gesture recognition system that achieves gesture recognition in different locations and different environments. However, such schemes usually rely on high-density deployments, which is impractical for large-scale deployments. The transmit–receive end is poor in mobility. When the position of the person changes, the position and orientation of the person need to be located first according to the send-receive link. On the basis of this, the cumulative error usually occurs. However, the mobile terminal can solve these problems very well. When the person moves, the receiving end can change according to the change of the person’s position. For example, WiGest [16] is based on the RSSI of the mobile terminal. However, WiGest only uses RSSI, which greatly limits its gesture recognition accuracy and resistance to environmental interference. According to the latest nexmon, our system can extract 234 CSI subcarriers on the mobile phone side and has a strong location and environmental adaptability while ensuring high recognition accuracy.

## 3. System Design

**Figure 1:**
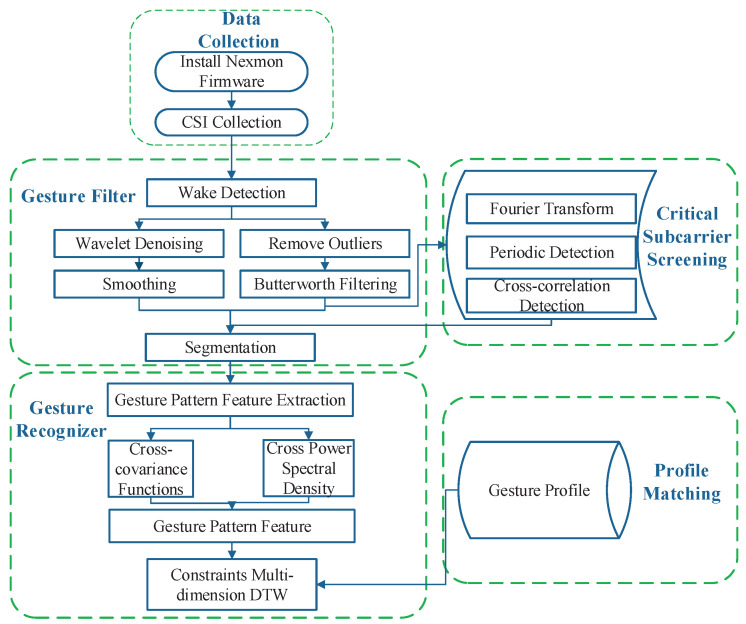
MDGest architecture.

Figure 1 shows the architecture and workflow of MDGest. The collected CSI signals are processed by three key components: gesture filter, critical subcarrier screening, and gesture recognizer. The signals not related to gesture operations are filtered out through gesture filter.. Simultaneously, after a gesture operation is detected, the MDGest system will segment the signal so that each segment corresponds to a gesture input. The gesture recognizer performs a recognition operation. The algorithm extracts the main mode features of each group of gestures, stores them in the form of a configuration file, and uses an improved DTW algorithm that can adapt to the individual differences in the speed and duration of gesture operations not synchronized to match pre-stored profiles.

### 3.1. Data Collection

We implemented MDGest on Android smartphones (Nexus 5) and commercial routers. We turn on monitor mode by installing the nexmon firmware on Nexus 5 phones, accessing the physical layer transmission information, and store physical layer CSI by creating new UDP frames. Then, we upload them to the host, using a Nexus 5 mobile phone to collect CSI with a bandwidth of 80 MHz in the 5 GHz of the commercial router. The total number of subcarriers collected can reach 256, and the number of available subcarriers is 234 after removing the guard bands, empty subcarriers, and pilot frequency signals. Comparing with gesture recognition using an Intel 5300 network card to collect only 30 subcarriers under a single link, MDGest dramatically improves the sensitivity and accuracy of gesture recognition.

CSI is the channel property of a communication link. It describes the shadow, multipath propagation, distortion and other information that wireless signals experience during propagation. Wireless channels can be described by channel frequency response in terms of amplitude–frequency characteristics and phase–frequency characteristics. The channel frequency response can be expressed using the following formula:

(1) $$H\left(k\right)=∥H\left(k\right)∥e^{j∠H(k)}.$$

H(k) represents the CSI of the subcarrier k.∥H(k)∥ and ∠H(k) are the amplitude response and the phase response of subcarrier k, respectively. In this paper, the proposed MDGest is based on the amplitude of the CSI.

### 3.2. Gesture Filter

The gesture filter removes signals not related to gesture operations and passes signals caused by gesture operations to the next stage. We set the wake-up operation part before gesture recognition starts. After the MDGest system recognizes the wake-up operation, it starts to perform the filtering operation. The wake-up operation can quickly wake up the system, thereby ensuring a low-energy sleep state to reduce energy consumption during the non-wake phase.

#### 3.2.1. Wake Detection

**Figure 2:**
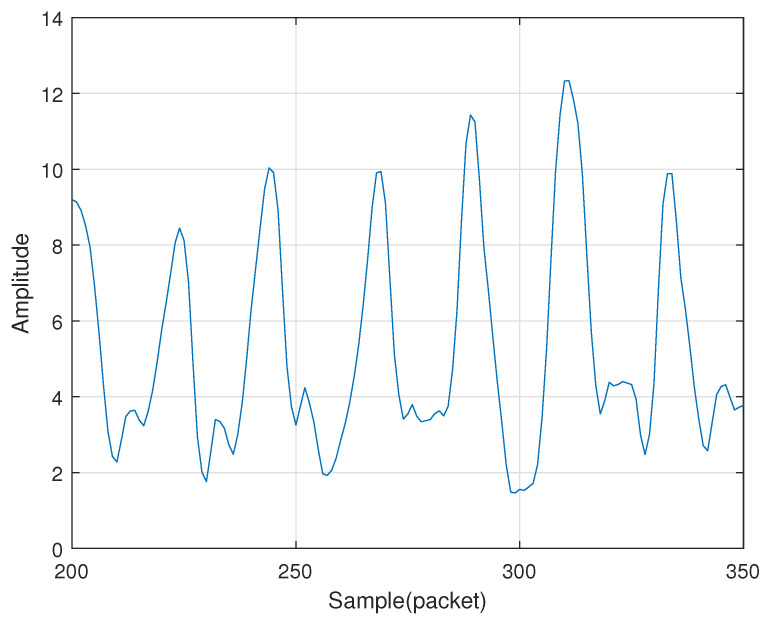
Rapid changes in amplitude.

To correctly determine when a user is performing a gesture, we set up a wake-up detection. The device is woken up when the user puts a hand near the receiver and then making n fast up and down gestures. Quick up and down gestures can cause rapid changes in energy amplitude. We detect the start of the wake-up action by setting a threshold and then start searching for n-1 periodic energy changes. In order to better set the adaptive threshold, we used a Nexus 5 mobile phone to perform multiple sets of up and down gesture calibration experiments at a fast speed. As shown in the Figure 2, the amplitude produced by the gesture shows an evident periodic change, and the peak value is about to be determined. Such periodic peak changes occur when the gesture begins to move up and down quickly. Therefore, MDGest uses this unique periodic amplitude change as a wake-up operation. In order to set a user-friendly wake-up operation, we chose a combination of up and down gestures with n of 4. In the experimental evaluation section, we will introduce the reasons for choosing 4.

#### 3.2.2. Filtering

**Figure 3:**
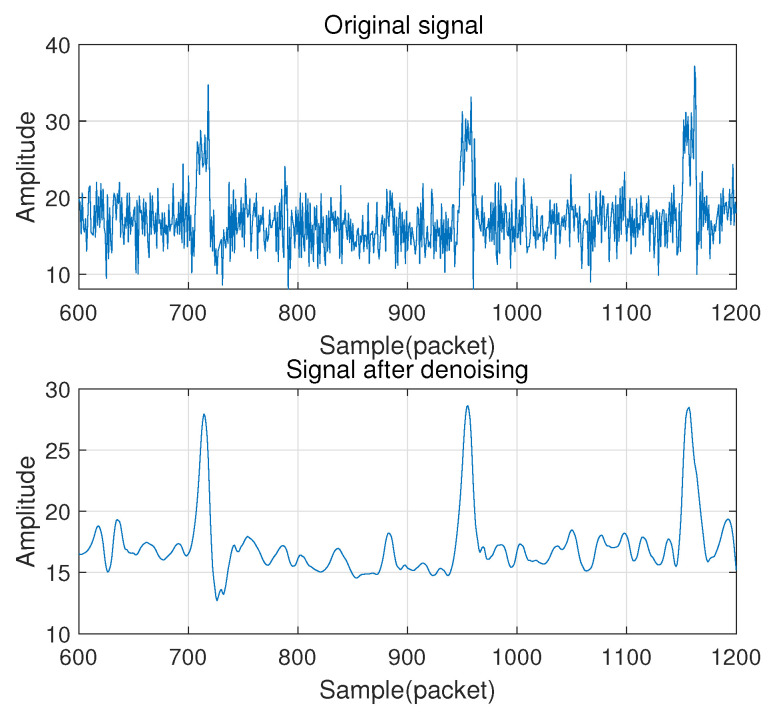
The original signal and the signal after filtering and denoising.

We first employ the Hampel algorithm to perform outlier removal on the original signal and then use a smoothing filter algorithm to smooth the signal. For each sample of the input vector, the Hampel function calculates the median of the window consisting of the sample and six surrounding samples, three on each side. The standard deviation of the median of each sample pair is estimated by using the median absolute value. If a sample is more than three standard deviations away from the median, the sample is replaced with the median. After that, a Butterworth bandpass filter is adopted to remove signals not related to gesture operations. Butterworth filter is chosen for bandpass filtering because it has the maximally flat frequency response in the passband and will not cause the largest distortion to the gesture signal. Finally, wavelet denoising is used to remove the residual noise components further while keeping the detailed patterns of the CSI. Figure 3 shows the original signal and the signal after filtering and denoising.

#### 3.2.3. Segmentation

**Figure 4:**
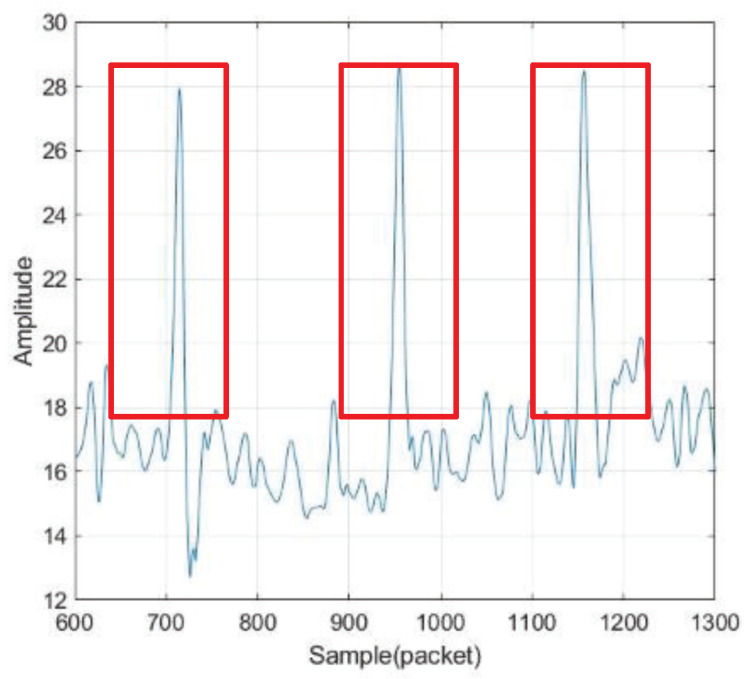
Segment the sample.

The system requires the user to have a short static interval after the wake-up action as a marker signal. We accumulate amplitude differences between adjacent time points. The cumulative value is then compared with the threshold value of the sentinel signal. We segment the gesture signal by detecting the two sentinel signals before and after. As shown in Figure 4, this algorithm is used to process the acquired CSI signals.

### 3.3. Key Subcarriers Selection

The selection of crucial subcarriers is mainly divided into the following steps: Fourier transform removes subcarriers whose frequencies are not in the frequency range of gesture recognition; periodic detection screens out subcarriers that do not correspond to the number of peaks and the number of gestures; cross-correlation detection filters out subcarriers with high correlation between different gestures. Through the screening of the above three links, we have selected the key subcarriers that are most sensitive to gesture recognition and assigned weights according to the degree of cross-correlation between these subcarriers for the gesture recognizer.

### 3.4. Gesture Recognizer

We expect that our system can not only perform gesture recognition at a fixed relative position to the transmitting end but also perform gesture recognition after the position changes of users, adapting to individual differences between different users. Therefore, we need unique characteristics to describe each gesture that a user operates.

#### 3.4.1. Gesture Mode Feature Extraction

Operating the same gesture, different users, or the same users will have different operating speeds and motion amplitudes, and the change of the users position will also cause the same gesture CSI to appear differently. To remove these individual and positional differences, we use Cross Covariance Function (CCF) and Cross Power Spectral Density (CPSD) to extract the main mode features of gestures. The CCF is defined as follows:

(2) $$R_{xy}\left(t_{1},t_{2}\right)\overset{\;\mathrm{def}\;}{=}E\left\{x\left(t_{1}\right)y^{*}\left(t_{2}\right)\right\}.$$

(3) $$\begin{array}{cc} C_{xy}\left(t_{1},t_{2}\right)\overset{\;\mathrm{def}\;}{=} & E\left\{\left[x\left(t_{1}\right)-\mu_{x}\right]{\left[y\left(t_{2}-\tau \right)-\mu_{y}\right]}^{*}\right\}\\ \; & =R_{xy}\left(\tau \right)-\mu_{x}\mu_{y}^{*}, \end{array}$$

where $R_{xy}$ and $C_{xy}$ are Cross Correlation Function and Cross Covariance Function of *x* and *y*, which are two CSI measurements. *u* is their mean value. τ represents the time difference between the two CSI measurements, called lag. The CCF can extract the common parts between the two CSI measurements and suppress the non-common parts. We perform CCF calculations on the CSI measurements of the same gesture to extract common parts.

The CCF involves a multiplication between two different CSI measurements. These two CSI measurements minus the mean have a common part and a non-common part, and the multiplication of the common part always takes the same sign, so that the part is strengthened and retained. In contrast, the non-common parts of the two CSI measurements are random. Their products are sometimes positive and sometimes negative. After the average operation of mathematical expectations, they tend to “cancel” each other. This means that the CCF can extract the common parts between the two CSI measurements and suppress the non-common parts. We perform CCF calculations on the CSI measurements of the same gesture to extract common parts.

The CCF describes the statistical properties of the CSI measurements in the time domain. However, it belongs to the time domain characteristic and cannot reflect the change of the gesture in the frequency domain. So we introduced CPSD, which describes the statistical properties of CSI measurements in the frequency domain.

The CPSD is defined as the Fourier transform of the CCF, as follows:

(4) $$P_{xy}\left(f\right)=\int_{-\infty }^{\infty }C_{xy}\left(\tau \right)e^{-j2\pi f\tau }d\tau .$$

where $P_{xy}\left(f\right)$ is the CPSD. The real part of CPSD is called the in-phase spectrum, and the imaginary part becomes the orthogonal spectrum, which is recorded as:

(5) $$P_{xy}\left(f\right)=\left|P_{xy}\left(f\right)\right|\exp\left[j\phi_{xy}\left(f\right)\right].$$

(6) $${\dot{\phi }}_{xy}\left(f\right)=\frac{d}{df}\phi_{xy}\left(f\right).$$

where $\left|P_{xy}\left(f\right)\right|$ and $\phi_{xy}\left(f\right)$ respectively represent the amplitude and phase of CPSD; ${\dot{\phi }}_{xy}\left(f\right)$ is called group delay.

After calculating and intersecting CCF and CPSD of the CSI, the primary mode characteristics of the same gesture signal in the time and frequency domains are obtained and stored in a library.

#### 3.4.2. Operation Identification

The MDGest system uses an improved DTW algorithm to classify gestures. DTW algorithm is a nonlinear warping method that combines time warping and distance measurement. The DTW algorithm calculates the minimum distance for two time series of different lengths. The smaller the distance between the two-time series is, the more similar the two-time series are.

When using the DTW algorithm for classification, it is necessary to test the distance between gesture sequences and template gesture sequences stored in the library. The category of the template sequence with the smallest distance is the gesture category of the test sequence.

The description of the test sequence and template sequence is shown in Equations (6) and (7):

(7) $$E\left(M\right)=\left\{e_{1},e_{2},\cdots ,e_{M}\right\}.$$

(8) $$H\left(N\right)=\left\{h_{1},h_{2},\cdots ,h_{N}\right\}.$$

E(M) and H(N) respectively represent test sequences of *M* data points and template sequences of *N* data points after feature extraction and data processing. Euclidean distance was used to calculate the distance between two sequence data points. The distance between the ith data $e_{i}$ of the test sequence and the jth data $h_{j}$ of the reference template sequence was calculated as shown in Equation (8):

(9) $$d\left(e_{i},h_{j}\right)=\sqrt{\sum_{l=1}^{L}{\left(e_{il}-h_{jl}\right)}^{2}}.$$

We calculate the Euclidean distance of the test sequence and the template sequence of data points to create a M×N distance matrix *D*. The value $\left(e_{i},h_{j}\right)$ of the element $d(e_{i},h_{j})$ represents the Euclidean distance of the data point $e_{i}$ and $h_{j}$. We use the DTW algorithm to calculate the distance between the two sequences, essentially looking for a suitable regular function j=f(i), and satisfy the function type (9):

(10) $$P\left(E,H\right)=\min_{f(i)}\sum_{i=1}^{M}d\left(e_{i},h_{f(i)}\right).$$

P(E,H) is the optimal matching distance between the test sequence E(M) and the template sequence H(N). The principle of the DTW algorithm is that the matrix *D* is to find a path from the starting point $\left(e_{1},h_{1}\right)$ to the ending point $\left(e_{M},h_{N}\right)$, and the cumulative distance of the path is the smallest. In order to achieve the optimal cumulative distance, the warping function of the DTW algorithm needs to meet the constraints of the global constraint and the local constraint. Among them, the local constraints that the warping function needs to satisfy are as follows:

(1) End point constraint, which requires that the starting point and ending point of the two sequences are consistent.

(11) $$\left\{\begin{array}{c} f(1)=1\\ f(N)=M \end{array}\right..$$

(2) Monotonic constraint. The generation of gesture data has a sequence, and the regularization function must ensure that the matching path does not violate the generation chronological sequence of gesture data, so it must satisfy Equation (11).

(12) f(n+1)⩾f(n).

(3) Constraint of continuity. In order to ensure the minimum loss of matching information, the warping function cannot skip any matching point. Solving the optimization problem through the DTW algorithm, the cumulative distance of the best path can be obtained as:

(13) $$p\left(\;e_{i},h_{j}\right)=d\left(\;e_{i},h_{j}\right)+\min\left\{\begin{array}{c} p\left(\;e_{i}-1,h_{j}-1\right)\\ p\left(\;e_{i}-1,h_{j}\right)\\ p\left(\;e_{i},h_{j}-1\right) \end{array}\right..$$

Among them, $p(e_{i},h_{j})$ represents the minimum cumulative distance of the path sought from point (1,1) to point (i,j) of matrix *D*, so the value of $p(e_{M},h_{N})$ is the minimum cumulative distance of the test sequence E(M) and the template sequence H(N), that is, the DTW distance between the two.

When the classic DTW algorithm is used to calculate the distance between two sequences, the DTW method requires calculation and storage of a larger matrix, and the calculation time complexity is O(mn). In order to improve the calculation efficiency of the DTW algorithm to calculate the distance of the gesture sequence, we introduce a global constraint window in the sequence bending calculation to avoid invalid path searching. The Constraints Multi-dimension Dynamic Time Wrapping (CM-DTW) algorithm uses the Sakoe–Chiba window to reduce the calculation of the distance between invalid data points [45,46], thereby improving the efficiency of calculating the two sequences.

The Sakoe–Chiba global constraint can be understood as a restriction on the subscript in the point $\left(e_{i},h_{j}\right)$, so that i−f⩽j⩽i+f is satisfied, and *f* is a constant.

Under the Sakoe–Chiba constraint, the two sequences of the test sequence E(M) and the template sequence H(N) calculate the matching and regular paths of the DTW distance as $c_{1},c_{2},\ldots ,c_{K}$ (where $c_{k}=\left(i,j\right)$ ).

Since the length of the gesture data sequence may vary greatly, it is necessary to make certain constraints on the slope of the search path so that the subscript satisfies:

(14) $$\frac{N*i}{M}-f⩽j⩽\frac{N*i}{M}+f.$$

The CM-DTW we adopted overcomes the problem of different gesture speeds and improves the calculation efficiency of DTW algorithm to calculate the distance of the gesture sequence, thereby providing a reliable measurement method for detecting the degree of matching between the CSI mode and multiple subcarriers.

## 4. Evaluation

**Figure 5:**
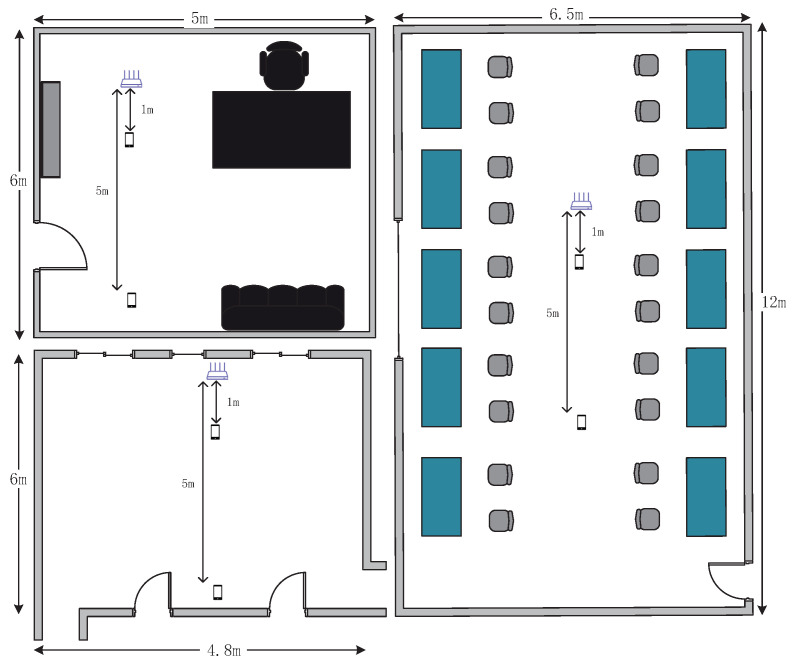
Layouts of three evaluation environments.

We evaluated the accuracy and robustness of MDGest based on the distance between the receiving and transmitting ends, the distance between users and equipment, the direction of users, and the environments. A total of 16 volunteers were recruited, including six females and ten males, aged between 20 and 28. The experiments were conducted in three indoor environments, including a laboratory, a large hall, and an office with furniture such as tables and sofas. Figure 5 shows the environmental characteristics of different rooms. The laboratory is 6.5 m × 12 m. There are experimental desks and chairs on both sides of the wall, and the middle corridor is empty. The hall is 6 m × 4.8 m, and it is empty. The office is 6 m × 5 m. There is a set of office desks, chairs, sofas, bookcases and so on. As shown in Figure 5, we placed a router and a Nexus 5 phone in the empty space of three rooms for data collection.

### 4.1. Implementation and Experimental Setup

We implemented the MDGest system on a Google Nexus 5 smartphone, which runs on Android 6.0. Furthermore, its firmware is modified with nexmon [21]. A TP-LINK TLWDR5620 wireless router is set as an AP. The AP possesses four omnidirectional antennas, two at 2.4 GHz and two at 5 GHZ. We set the AP to IEEE 802.11ac mode at 5.21 GHz (channel number 42) on 80 MHz bandwidth. We set the Nexus 5 smartphone as MP and turn on its monitor mode through the nexmon firmware. The nexmon firmware can access the physical layer transmission information, and store physical layer CSI by creating new UDP frames. Then, it will upload them to the host. All collected CSI are eventually stored in a PCAP file containing 256 complex pairs. However, the number of available subcarriers is 234 after removing the guard bands, empty subcarriers, and pilot frequency signals. The prototype system is implemented in MATLAB.

We fixed the position of the router at one end of the room, and the height was about 1 m, which is accorded with the typical indoor scene. Then, we used a Nexus 5 smartphone to collect gesture data at different distances and directions from the router. The types of gestures collected include push-pull, sweep, clap, slide, circle, and zigzag. Sixteen users collected 2160 gestures over three weeks, including different rooms, distances, and directions.

There is no fixed execution time for each gesture. The timing of each gesture depended on the habits of the volunteers. This makes the system more robust to users with different gesture execution speeds. In each scenario, each user continuously collects the same gesture for 20 times, and there is a 2–3 s static interval between each time, so that the system can better divide the data.

### 4.2. Overall Accuracy

**Figure 6:**
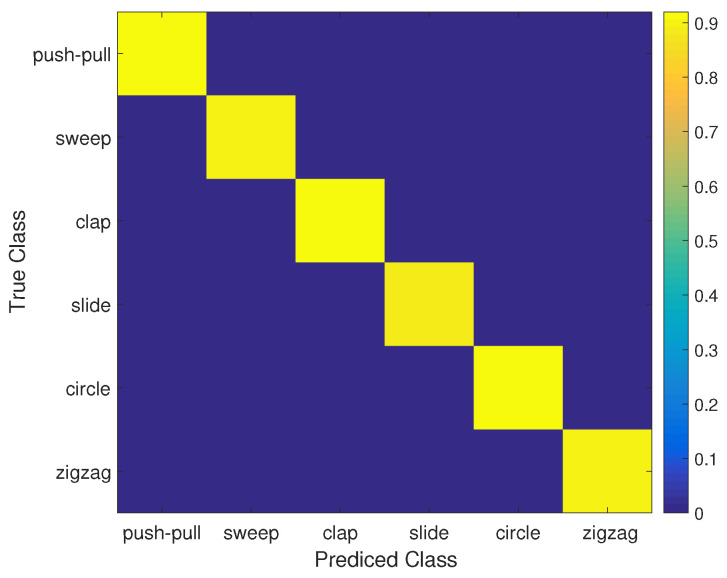
Confusion matrix of different operations.

**Figure 7:**
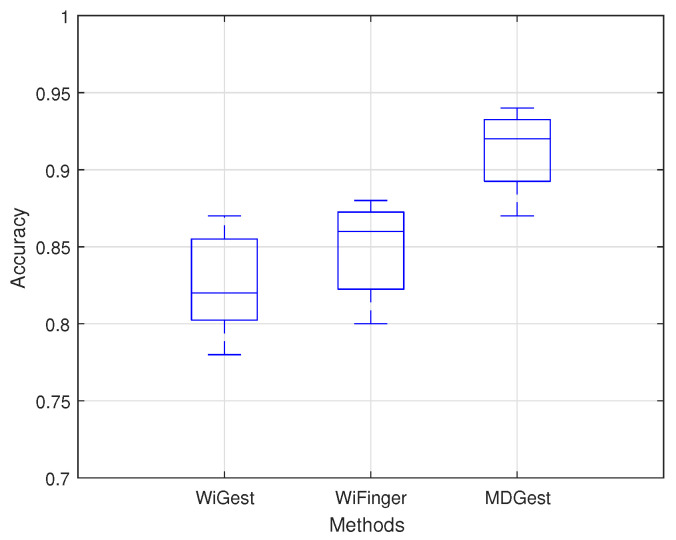
Accuracy of different methods.

**Figure 8:**
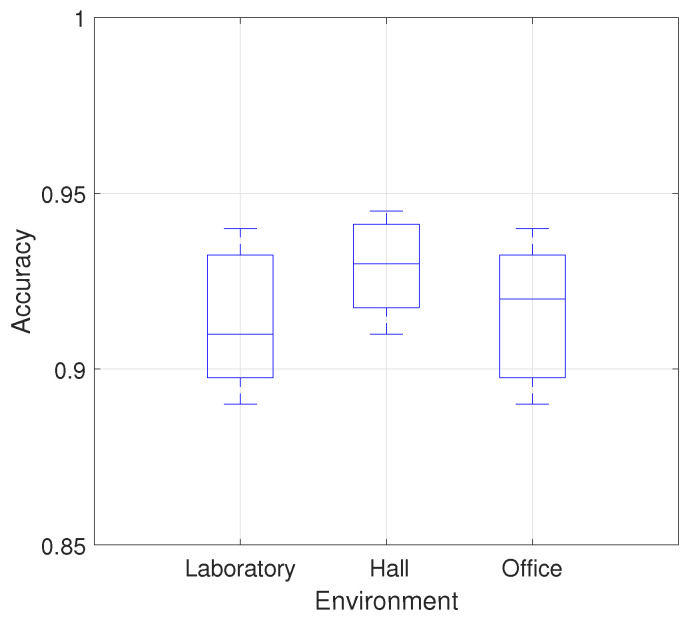
Accuracy of different environments.

The overall recognition accuracy of six gestures in three environments is shown in Figure 6 and Table 1, which indicates that the overall average accuracy of the MDGest system reaches 92%. Furthermore, we compared MDGest with WiGest and WiFinger, both gesture recognition systems using only one transmitter and one receiver. WiGest [16] is based on RSSI extracted from a smartphone and using Discrete Wavelet Transform for frequency feature extraction. WiFinger [9] is based on CSI extracted from a laptop equipped with an Intel 5300 network card, using Dynamic Time Warping for waveform comparisons. We compared the three approaches in a typical office environment. The Figure 7 results show that our system can provide high accuracy of gesture recognition in the three systems. Moreover, we also evaluated the performance of MDGest system in different environments. The Figure 8 shows the average recognition accuracy of the system in laboratory, hall, and office environments. The results show that the MDGest system can maintain an average accuracy of more than 90% in three different environments.

### 4.3. Impact Factors

Since we are using a smartphone as the receiving end, we have to consider changes in the relative position of the receiving end and the transmitting end, such as distance, angle, etc. In addition, changes in the distance, direction, and environment between users and equipment also affect system recognition performance. We also consider the false detection rate caused by environmental factors in the absence of gestures. Multiple factors have been taken into consideration from distance, over orientation to environmental disturbance, which we think could affect the recognition accuracy of the system.

#### 4.3.1. Impact of Distance Between Transmitting and Receiving ends

Since the location of the smartphone changes with the user, it is essential that the performance of the system remains stable at different distances from the smartphone to the AP. We evaluated the accuracy under the distances from 1 m to 5 m in 0.5 m steps between the transmitter and receiver. We fixed the distance between the user and the smartphone at 1 m.

**Figure 9:**
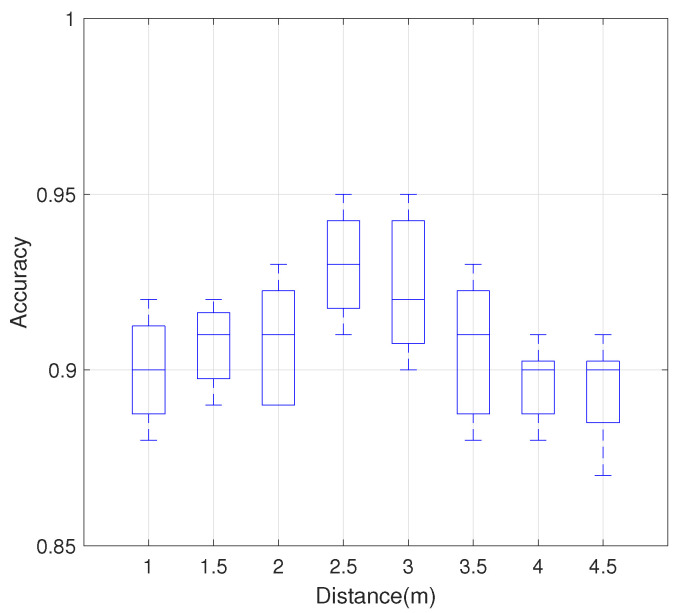
Impact of distance between transmitting and receiving ends on the recognition accuracy.

As shown in Figure 9, the overall accuracy of the system is maintained above 90%. In the case of distances of 2.5 m and 3 m, the recognition accuracy of the system reaches up to 95%, which is different from our common sense that the closer the receiver and transmitter are, the higher the accuracy of gesture recognition is. We guess that this may be due to the fact that the CSI signal of the direct view path cannot fully cover all the motion information of the gesture when the distance between the receiving end and the transmitting end is too close.

#### 4.3.2. Impact of Distance between Hand and Device

When users perform gestures, the same gesture will lead to different CSI changes at different distances from the smartphone. Therefore, it is vital to evaluate system performance at different distances from the smartphone. The distance between the smartphone and the AP is set to 2.5 m. The user performs gestures in the direct path between smartphone and AP under distances from 20 cm to 160 cm in 20 cm steps between hand and smartphone.

**Figure 10:**
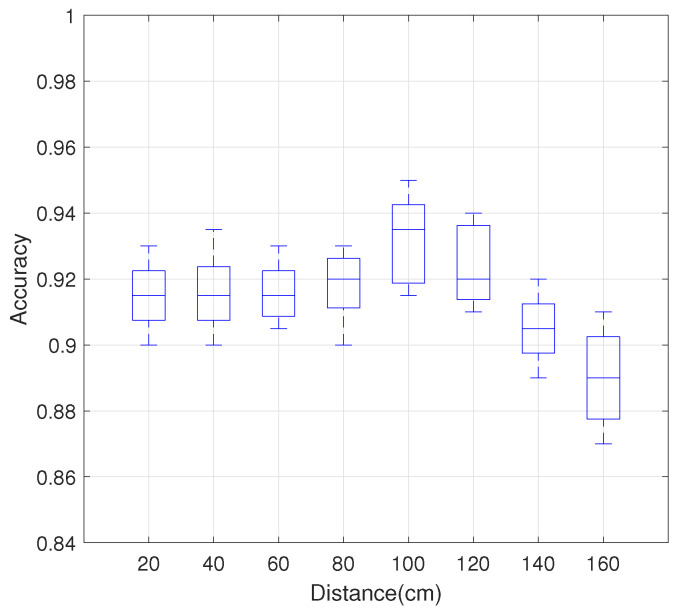
Impact of distance between hand and device on the recognition accuracy.

Figure 10 shows the relationship between MDGest system’s gesture recognition accuracy and the distance of the hand relative to the device. As the distance between the hand and device increases, the accuracy of recognition also increases, reaching an accuracy of 95% at 1 m. This is very intuitive. As the distance of the gesture from the device increases, the amplitude of the edge of the gesture also increases, resulting in a better signal-to-noise ratio and more accurate gesture detection. As the distance increases further, the accuracy decreases, but the overall accuracy remains 90%.

#### 4.3.3. Impact of User Orientation around the Device

**Figure 11:**
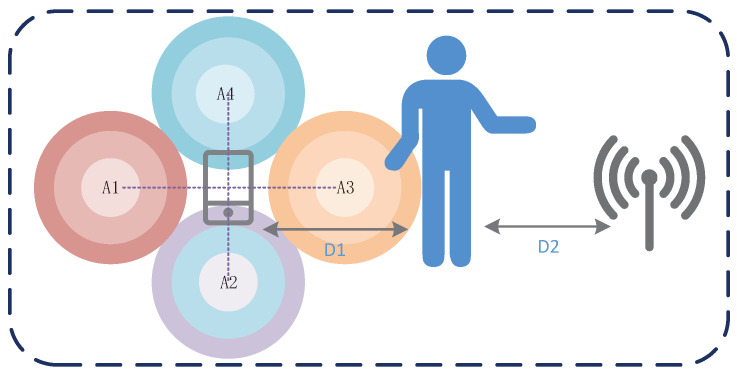
Different positions and regions of gestures input.

Although the user is in the direct path of the smartphone and router by default when performing gestures, we still evaluate the performance of the system when the user performs gestures in other relative areas of the device. In this section, we show the effect of the user performing gestures in different orientation areas around the device on the accuracy when the device’s position and orientation are fixed. We evaluated the performance of the user in four directions of the device, which is shown in Figure 11. The center of region A1, A2, A3, and A4 is 1 m away from the smartphone center. The distance between the mobile device and the AP is set to 2.5 m.

**Figure 12:**
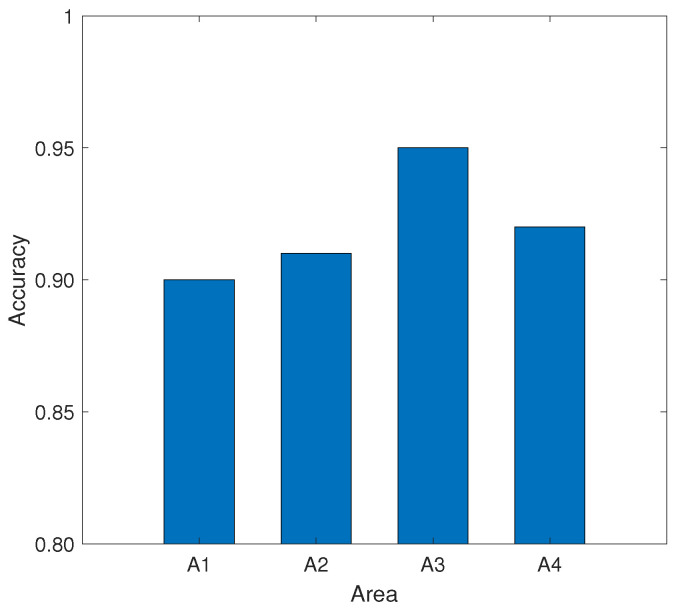
Impact of input region on recognition accuracy.

As shown in Figure 12, the recognition accuracy for the four regions is all above 90%, which indicates the MDGest system can maintain high accuracy in the surrounding areas. Among the four regions, region A3 has the highest recognition accuracy because the location of region A3 is exactly in the direct path of smartphone and AP, and gesture motion information can be better captured by CSI. In contrast, the other three regions can only reflect signals by the gesture to cause CSI changes. Therefore, the identification accuracy is lower than the region A3.

#### 4.3.4. Impact of Environmental Interference

The MDGest system realizes gesture recognition depending on the changes of CSI over the LOS path. Therefore, the gesture recognition accuracy of the system is seriously affected by the interference of people in or near the LOS path. Considering the existence of human interference in the real environment, we evaluated the robustness of the system to humans disturbances. We set the distance between the receiver and the transmitter to 2.5 m. The ambient interference we set up is an interfering person walking at different distances from the transceiver link’s vertical line.

**Figure 13:**
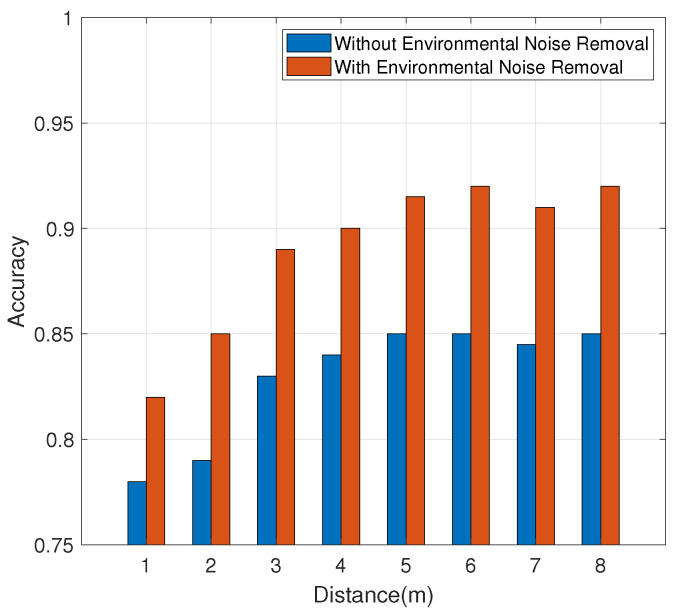
Impact of environmental interference.

Figure 13 reflects the results, showing that when the distance of the interference user is less than 3 m, the accuracy will be seriously affected. When the interferer is more than 4 m away, the accuracy will be reduced in the absence of denoising. After the removal of environmental noise, the impact on accuracy is insignificant. In general, the system can maintain high accuracy after noise removal.

#### 4.3.5. False Detection Rate Without Gestures

As mentioned in Section 3.2.1, MDGest uses unique gestures with periodic changes as wake-up operations to reduce energy consumption. We evaluated the false detection rate of the MDGest system in the absence of actual gestures. We conducted experiments in the laboratory. There were seven students in the room for daily activities. There were other visitors coming in and out during the period, and the number of people in the room can reach up to 10 at most.

**Figure 14:**
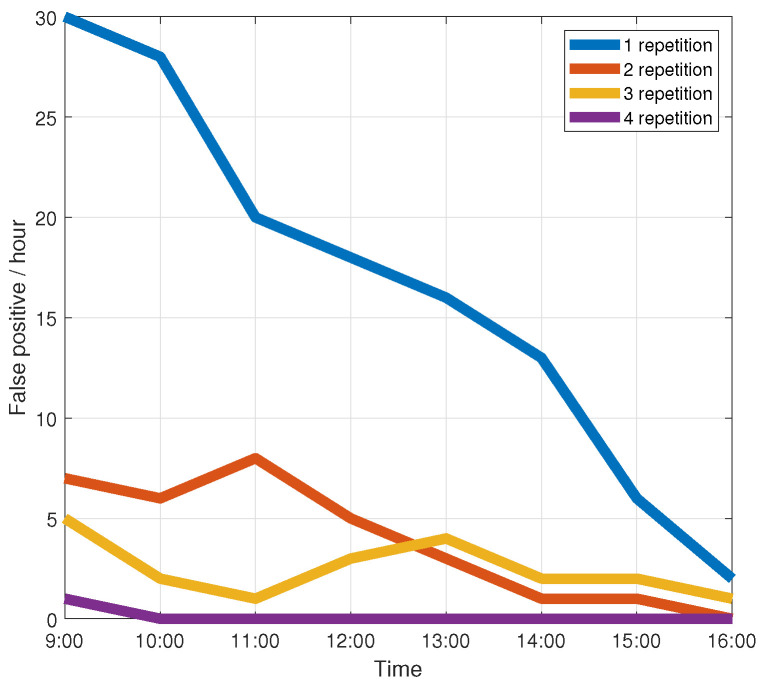
False detection rate in the absence of actual gestures.

Figure 14 shows the average false detection rate per hour. We have observed that accuracy increases as the number of wake-up actions increases. When the number of wake-up actions is 4, it can reach about 0 error detections per hour. Nevertheless, four wake-up actions are too tedious for the user. Figure 14 shows that when the number of wake-up actions reaches three, the average false detection rate per hour can reach 3.4, which can meet the requirements of the system. So we used three wake-up actions in the paper.

## 5. Discussion

### 5.1. Why Use Smartphones for Gesture Recognition?

First, when we use a PC equipped with Intel 5300 network card or Atheros 9580 network card to recognize gestures, we need to walk to a certain range near the computer to get accurate gesture recognition. This is necessary because the location of the executing gesture must be in at least one Los link so that the WiFi signal can accurately capture the signal changes caused by the gesture. It is difficult to achieve high recognition accuracy for gestures that are completely out of the Los link. However, in daily life, almost everyone carries a smartphone, so we can take out the smartphone for gesture recognition at any time, instead of preparing a laptop in advance and walking near the laptop or bringing it to us for gesture recognition. When we take out the phone, we can easily put ourselves in the Los link near the phone. From this point of view, we believe that the advantages of smartphones are far greater than laptops. Second, supporting 802.11ac and allowing for bandwidths of up to 80 MHz is a huge advantage. Using the nexmon CSI extraction tool can extract up to 256 subcarriers, which is currently only possible with 802.11ac and 80 MHz bandwidth.

### 5.2. The Limitations of Nexmon Firmware

The current nexmon firmware is applicable to smartphones, Raspberry Pi B3+/B4, and Asus RT-AC86U with Broadcom bcm43xx series chips. For smartphones, the nexmon firmware can support many smartphone brands, including Samsung, Apple, Huawei, Google, SONY, etc. However, only some of these smartphone brands are equipped with Broadcom bcm43xx series WiFi chip. In addition, there are many other smartphone brands that cannot be supported. The previous Linux 802.11n CSI Tool [14] and Atheros CSI Tool [15] are in cooperation with Intel and Qualcomm to obtain CSI. Nexmon firmware was developed through reverse engineering of Broadcom bcm43XX series WiFi chips with open-source firmware, so if nexmon wants to support more smartphones, it will need more WiFi chip vendors with open-source firmware. However, we believe that with the rapid development of WiFi-based wireless sensing technology, more and more chip manufacturers will expand the opening of the underlying CSI of the chip.

### 5.3. The Energy Consumption Problem

Realizing gesture recognition on smartphones can provide users with a more convenient gesture recognition experience. However, the problem of energy consumption will still bring great trouble to users. The main energy sources of the MDGest system in smartphone deployment are from two aspects. One is that the phone needs to receive the packet all the time and determine whether it is a gesture performed by the user. The other is to run feature extraction and recognition algorithms. In the former case, we have reduced some energy consumption by setting up a wake-up detection method. For the latter, we will further optimize the feature extraction and recognition algorithm in future work to reduce the computational complexity and reduce energy consumption.

## 6. Conclusions

In this paper, we presented MDGest, a WiFi-based single-link gesture recognition system that employs CSI extracted from smartphones. MDGest leverages nexmon firmware to extract 234 CSI subcarriers from the smartphone in IEEE 802.11ac mode at 5.21 GHz (channel number 42) on 80 MHz bandwidth. Then MDGest extracts time domain and frequency domain features and exploits the improved CM-DTW algorithm to recognize the gestures. We implemented MDGest on off-the-shelf consumer smartphones and conducted comprehensive experiments. Experimental results show that MDGest can maintain high recognition accuracies across different environments, users, and locations. MDGest is robust to environmental and individual differences.

In future work, we will invite volunteers of different ages to participate in the experiment, so as to expand the coverage of experimental data and improve the robustness of the system to different age groups. Furthermore, we will continue to optimize feature extraction and gesture recognition algorithms to reduce algorithm complexity and system operation energy consumption.

## Figures and Tables

**Table 1:** The average recognition accuracy of each gesture in the three environments.

*EG*	Push-Pull	Sweep	Clap	Slide	Circle	Zigzag
Laboratory	94.7%	89.5%	95.7%	87.0%	94.8%	88.4%
Hall	96.2%	90.3%	94.5%	89.5%	96.0%	89.1%
Office	94.1%	90.8%	92.4%	89.2%	95.0%	87.0%
